# *CSNK2B*-associated epilepsy: family report and literature review

**DOI:** 10.1186/s42494-026-00278-y

**Published:** 2026-09-20

**Authors:** JiaLi Zhang, YueLin Li, YuRong Xiong, ShuJing Pan, LiJuan Yang, RenKe Li, MaoQiang Tian

**Affiliations:** 1 Department of Pediatrics, Affiliated Hospital of Zunyi Medical University, Zunyi, 563000 China; 2 Department of Pediatrics, Guizhou Children’s Hospital, Zunyi, 563000 China; 3 Guanling Buyi and Miao Autonomous County People’s Hospital, Anshun, 561300 China

**Keywords:** *CSNK2B*, Poirier-Bienvenu neurodevelopmental syndrome, Epilepsy, Drug-resistant epilepsy, Genotype–epilepsy phenotype correlation

## Abstract

**Background:**

*CSNK2B* mutations are associated with Poirier-Bienvenu neurodevelopmental syndrome (POBINDS), in which epilepsy is a common clinical feature. To describe the epilepsy phenotype of *CSNK2B* mutations, we reported a family with a novel *CSNK2B* mutation, added this information to the existing literature, and then combined these data for analysis to offer some observations.

**Methods:**

We performed trio-based whole-exome sequencing and Sanger validation in a family with early-onset seizures. In addition, we reviewed published reports of patients carrying pathogenic or likely pathogenic *CSNK2B* mutations, extracted epilepsy phenotypic data, and analyzed the correlation of epilepsy severity with the mutation type and the mutation location within the protein (specific amino acid position or domain).

**Results:**

We identified a novel pathogenic nonsense mutation in *CSNK2B*, c.240T > G, p. Tyr80*, among three affected members of one family. Together with published cases, a total of 103 individuals with sufficient epilepsy-related data were available for analysis. Seizures occurred in 94.2% of patients, 87.4% were diagnosed with epilepsy, and seizure onset before 24 months of age in 87.2%. There were no significant differences in the incidence of seizures, epilepsy, and drug‑resistant epilepsy (DRE) among different mutation type groups (*P* = 0.313, 0.069, and 0.690, respectively). Subgroup analysis of missense mutations into conservative and radical substitutions also revealed no significant differences in epilepsy and DRE incidence (*P* = 1.000 and *P* = 0.714, respectively). In contrast, mutation location appeared to be more informative: mutations outside annotated protein domains showed higher epilepsy penetrance than those within domains (*P* = 0.034). Exploratory analyses also suggested that the distribution of mutations across different domains varies, which may affect epilepsy severity, though further validations are required.

**Conclusions:**

This study expanded the mutational and phenotypic spectrum of *CSNK2B*-related disorders by identifying a novel pathogenic nonsense mutation. Protein-level mutation location appeared to be more informative than mutation type in relation to epilepsy severity. We noticed that the composition of intradomain mutations may play a part in predicting DRE susceptibility. Further large-scale validation studies are in need.

**Supplementary Information:**

The online version contains supplementary material available at https://doi.org/10.1186/s42494-026-00278-y.

## Background

Casein kinase 2 beta gene (*CSNK2B*, OMIM*115441) encodes the β subunit of casein kinase 2 (CK2), a highly conserved serine/threonine kinase that participates in diverse biological processes including signal transduction, metabolism, replication, transcription, and translation. CK2 predominantly exists as a tetrameric complex consisting of two catalytic subunits (α and/or α′) and two regulatory β subunits [1, 2]. The human CSNK2B protein contains 215 amino acids and five annotated functional regions: a KEN box (named for the tripeptide K‑E‑N, residues 32–40), a destruction box motif (D-box, residues 47–55), an acidic region (A, residues 55–77), a zinc finger domain (Z, residues 105–140), and a CK2α-interaction region (residues 181–203) [3–7]. At present, mutations in *CSNK2B* have been reported to be associated with Poirier-Bienvenu neurodevelopmental syndrome (POBINDS, OMIM #618732), an autosomal dominant disorder. This syndrome, also known as “*CSNK2B* encephalopathy” or “*CSNK2B*-related disorders”, is a neurological disorder characterized by early-onset seizures and variably impaired intellectual development, with a highly heterogeneous clinical presentation [8, 9]. The reported clinical phenotype includes developmental delay, intellectual disability, epilepsy, hypotonia, growth abnormalities, and variable dysmorphic features. Among these manifestations, epilepsy is one of the most clinically significant phenotypes, with marked heterogeneity ranging from seizure-free, febrile seizures, and drug-responsive epilepsy to severe early-onset epilepsy or drug‑resistant epilepsy (DRE). This variability increases the complexity of clinical recognition and prognostic assessment [5, 10–12].

The *CSNK2B* gene is widely expressed across multiple tissues, with particularly high expression in the cerebral cortex and hippocampus, according to the Human Protein Atlas. In addition, *CSNK2B* mRNA expression is higher during early developmental stages (i.e., the embryonic and fetal periods) than during childhood and adulthood [13]. In animal studies, homozygous disruption of the *Csnk2b* gene results in post-implantation lethality, with mutant embryos showing reduced size, decreased cell proliferation, and developmental arrest as early as embryonic day 6.5. Moreover, heterozygous embryos also displayed reduced size and developmental delay at embryonic day 10.5 [14, 15]. The high expression of *CSNK2B* during early development, together with mouse evidence linking its dysregulation to embryonic abnormalities and the finding that *CSNK2B* mutations cause early‑onset epilepsy, suggests that disruption of the protein’s function may impair early neurodevelopmental processes.

Previous studies have analyzed genotype‑phenotype correlations in *CSNK2B*-related disorders through domain-level and mutation type analyses [5, 7, 8, 10–12, 16]. Nevertheless, because few patients carrying the same or similar *CSNK2B* mutations have been reported, the determinants of epilepsy heterogeneity remain incompletely understood. In this study, we identified a family carrying a novel *CSNK2B* nonsense mutation, NM_001320(*CSNK2B*): c.240T > G, p. (Tyr80*). Furthermore, we evaluated the epilepsy-related clinical features of our patient together with all previously reported *CSNK2B* mutation cases to delineate the clinical spectrum and to explore factors potentially associated with variability in epilepsy severity.

## Methods

### Subjects

Patients with unexplained epilepsy or familial febrile seizures were recruited from the Department of Pediatrics, Affiliated Hospital of Zunyi Medical University, between May 2015 and August 2025. The study adhered to the guidelines of the International Committee of Medical Journal Editors regarding patient consent for research or participation. This study was approved by the ethics committee of the Affiliated Hospital of Zunyi Medical University (Ethics Approval Number: KLL-2025-636). Written informed consent for genetic testing and publication of data was obtained from all parents. We collected and analyzed patients’ clinical information, including demographic information, the types and frequencies of seizures, neurological examination results, family history, response to antiseizure medications (ASMs), results of brain magnetic resonance imaging (MRI) and video electroencephalography (EEG). Epileptic seizures or epilepsies were diagnosed according to the criteria of the Commission on Classification and Terminology of the International League Against Epilepsy (ILAE). Developmental and epileptic encephalopathy (DEE) describes a condition in which underlying brain pathology, or developmental encephalopathy, combines with frequent epileptic seizures or epileptiform discharges, the epileptic encephalopathy, to cause severe impairment, regression, or stagnation of neurodevelopment [17]. Febrile seizures are seizures triggered by fever in children aged 6 months to 5 years without a history of an unprovoked seizure or concurrent central nervous system infection [18]. DRE was defined as failure to achieve sustained seizure freedom after adequate trials of two tolerated and appropriately chosen ASM regimens, either monotherapy or combination therapy. Because the term “benign epilepsy” is no longer preferred by the ILAE, we used the term “Non-drug‑resistant epilepsy” and defined it operationally as epilepsy in which seizures are well controlled with ASMs or remit spontaneously, with or without developmental regression, during follow-up[17,19].

### Trio-based whole-exome sequencing

Blood samples were obtained from the probands and their parents to determine the origin of the identified genetic mutations. Genomic DNA was extracted from peripheral blood using a QuickGene DNA Whole Blood Kit (Fujifilm, Tokyo, Japan). Exome capture was performed using the xGen Exome Research Panel (Integrated DNA Technologies, Coralville, IA, USA), and paired-end read sequences were generated on the NovaSeq 6000 sequencing system. Sequences were aligned to the human reference GRCh38 (hg38) genome assembly. The identified mutations were annotated with AnnoVar and evaluated based on allele frequencies, pathogenicity predictions, and protein function [20]. Pathogenic mutations relevant to clinical phenotypes were further verified by Sanger sequencing.

### Literature review and exploratory genotype-epilepsy analysis

To summarize the epilepsy-related phenotypes associated with *CSNK2B* mutations, we reviewed previously reported patients carrying pathogenic or likely pathogenic *CSNK2B* mutations with available detailed neurological phenotype data. The Human Gene Mutation Database (HGMD) was used as an initial search aid, and the corresponding original publications were consulted to verify mutation types and available clinical information. Patients were included if the reports provided sufficiently detailed information on at least one of the following: seizure history, epilepsy diagnosis, or response to ASMs. Duplicate reports and cases with unconfirmed mutation classification or insufficient phenotypic information were excluded. Duplicate records of the same patient across different publications were subsequently merged into one final record.

For exploratory analyses, mutations were classified as missense or non-missense. The non-missense group included nonsense, frameshift, splice-site, start-loss, deletion, in-frame, and other non-missense mutations. Additionally, we divided missense mutations into conservative and radical groups based on Grantham scores. The conservative group contained conservative and moderately conservative mutations, while the radical group contained moderately radical and radical mutations. Because the pathogenic mechanisms of *CSNK2B* mutations remain incompletely understood and the functional consequences may differ substantially across mutation types, we did not classify mutations simply as loss-of-function (LOF) or non-loss-of-function. All subgroup analyses were considered exploratory because of the small sample size of several categories and the literature-derived nature of the dataset.

### Statistical analysis

Statistical analyses were performed using SPSS version 29.0. Categorical variables were summarized as counts and percentages. Fisher’s exact test was used for comparisons between two groups. Comparisons involving sparse subgroups were interpreted descriptively. All tests were two-sided, and *P* < 0.05 was considered statistically significant. No adjustment for multiple comparisons was performed. Therefore, all inferential results should be interpreted cautiously.

## Results

### Identification of the *CSNK2B* mutation

**Fig. 1:**
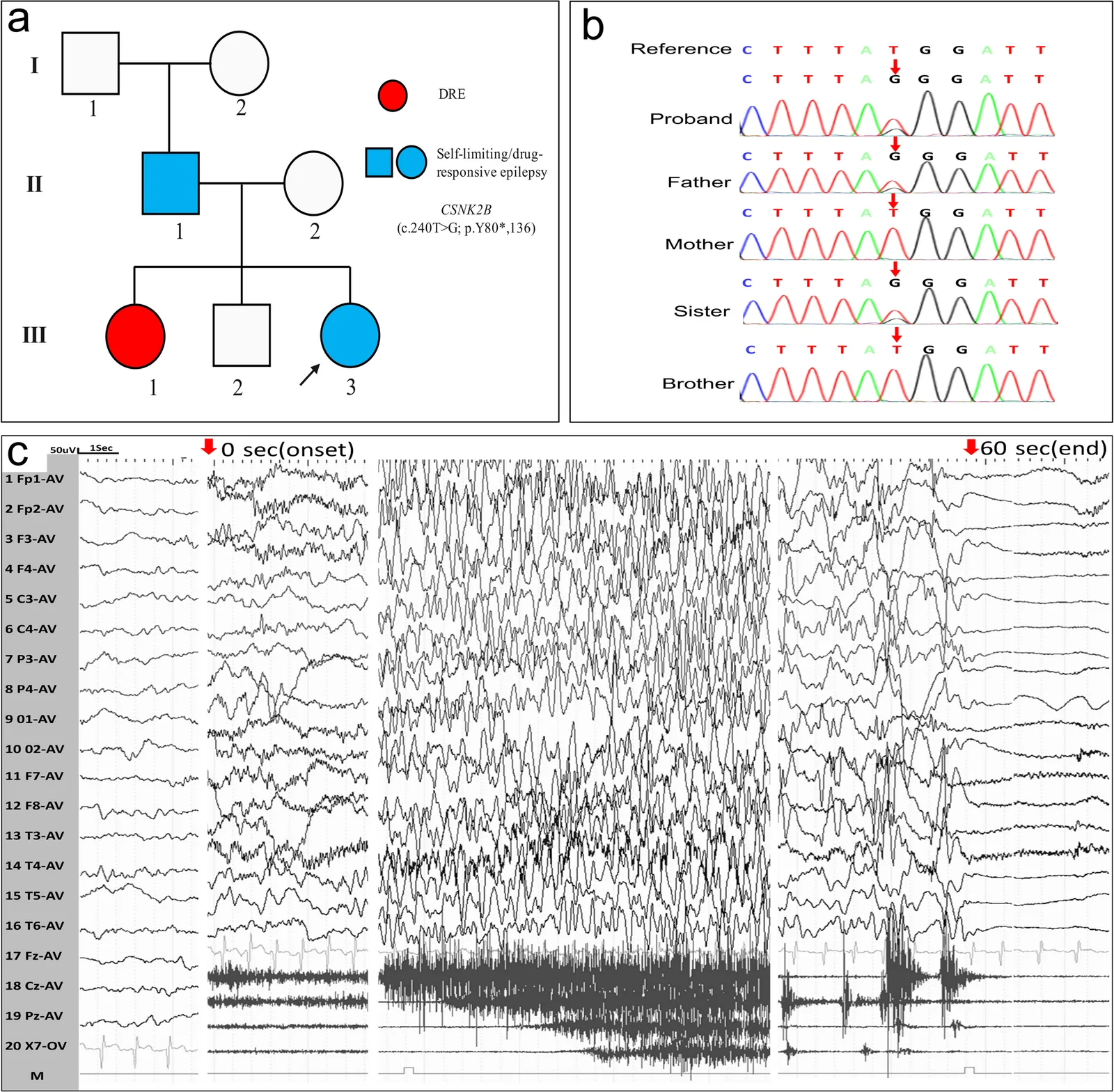
Clinical and genetic data on patients with the *CSNK2B* mutation. **a**. Pedigrees of the cases with the *CSNK2B* mutation. **b**. DNA sequence chromatograms of the *CSNK2B* mutation. **c**. Electroencephalography recordings of the proband revealed multifocal sharp and spike waves and captured two tonic-clonic seizures originating from the bilateral frontal regions during sleep (obtained at 3 months of age)

From a screening of 200 Chinese families with unexplained epilepsy, we identified one family with three affected individuals carrying the *CSNK2B* nonsense mutation c.240T > G, p. Tyr80* (Fig. 1a). This mutation was present in the proband, sister, and father, but not in the mother or brother (Fig. 1b). The mutation was absent from gnomAD v4.1.0, and the affected gene is highly conserved across multiple species [21]. According to the American College of Medical Genetics and Genomics (ACMG) criteria, it was classified as pathogenic (Table 1).

**Table 1:** Genetic Data of *CSNK2B* p.(Tyr80*) mutation

Case	Mutation (NM_001320)	Mutation Type	Mutation origin	MAF (gnomAD)^1^	CADD (GRCh37-v1.7)^2^	GERP + + RS^3,6^	phyloP^4,6^	ACMG Classification & Evidence^7^
1–3	c.240T > G p. (Tyr80*)	Nonsense mutation	Paternal inheritance^5^	Not found	36.0(Pathogenicity)	2.19 (Moderate)	0.448 (Weak)	**P**(PVS1 + PM2-Supporting+PP1)

*1.MAF from gnomAD v4.1.0. Absence supports PM2. 2.CADD PHRED score (GRCh37-v1.7). Scores > 20 suggest deleteriousness. 3.GERP + + RS score from UCSC (allHg19RS_BW). Positive scores indicate evolutionary constraint. 4.phyloP score from UCSC (phyloP100wayAll). Positive scores indicate evolutionary constraint. 5.Paternal origin: Present in the affected father; without grandparental testing, it is unknown if inherited or *de novo* in him. 6.Prediction Summary: High CADD supports deleteriousness. Moderate/weak conservation scores suggest the specific tyrosine is not maximally constrained, but its truncation by a nonsense mutation is predicted damaging (PVS1). 7.Pathogenicity Summary: Pathogenic classification is based on: (i) PVS1 (loss-of-function), (ii) PM2_Supporting (absent in controls), and (iii) PP1 (co-segregation)

### Clinical features of three patients

Three affected family members presented with early-onset seizures and had an overall favorable prognosis. The remaining family members had no history of seizures.

#### Case 1

**(III.3)**: Proband, a 2-year-old female. She presented with focal seizures evolving to bilateral tonic-clonic seizures, characterized by impaired consciousness, fixed gaze, facial cyanosis, and limb movements at 3 months of age. The initial seizure frequency was 5–6 episodes per day. Development was normal prior to onset and remained age-appropriate. Examinations and brain MRI were normal. EEG showed diffuse slowing and multifocal epileptiform discharges, with recorded seizures originating frontally (Fig. 1c). Seizures were fully controlled on a combination of oxcarbazepine (OXC, 33.3 mg/kg/day) and levetiracetam (LEV, 55.6 mg/kg/day). At the last follow-up (2 years 7 months old), she had remained seizure-free for more than 2 years and had normal development.

#### Case 2

**(III.1)**: A 13-year-old female. Neonatal onset of focal seizures with impaired awareness, head deviation, and automatisms (e.g., swallowing) were observed. Seizures lasted 2–3 min and occurred several times per month. Valproic acid (VPA) monotherapy had been ineffective. Seizures persisted into adolescence, possibly in the setting of poor medication adherence. After a recent consultation, OXC (20 mg/kg/day) was added to the regimen, with gradual dose titration planned. During 4 months of follow-up, seizure frequency decreased to 1–2 episodes per month and seizure duration shortened. Motor and language development were normal, but she had mild intellectual impairment with learning difficulties.

#### Case 3

**(II.1)**: The father had a history of seizures beginning at 6 months of age, with remission after approximately 4 years. Details of seizure semiology and treatment were unavailable. Psychomotor development was reportedly normal.

### Epileptic phenotype of patients with *CSNK2B* mutations

**Fig. 2:**
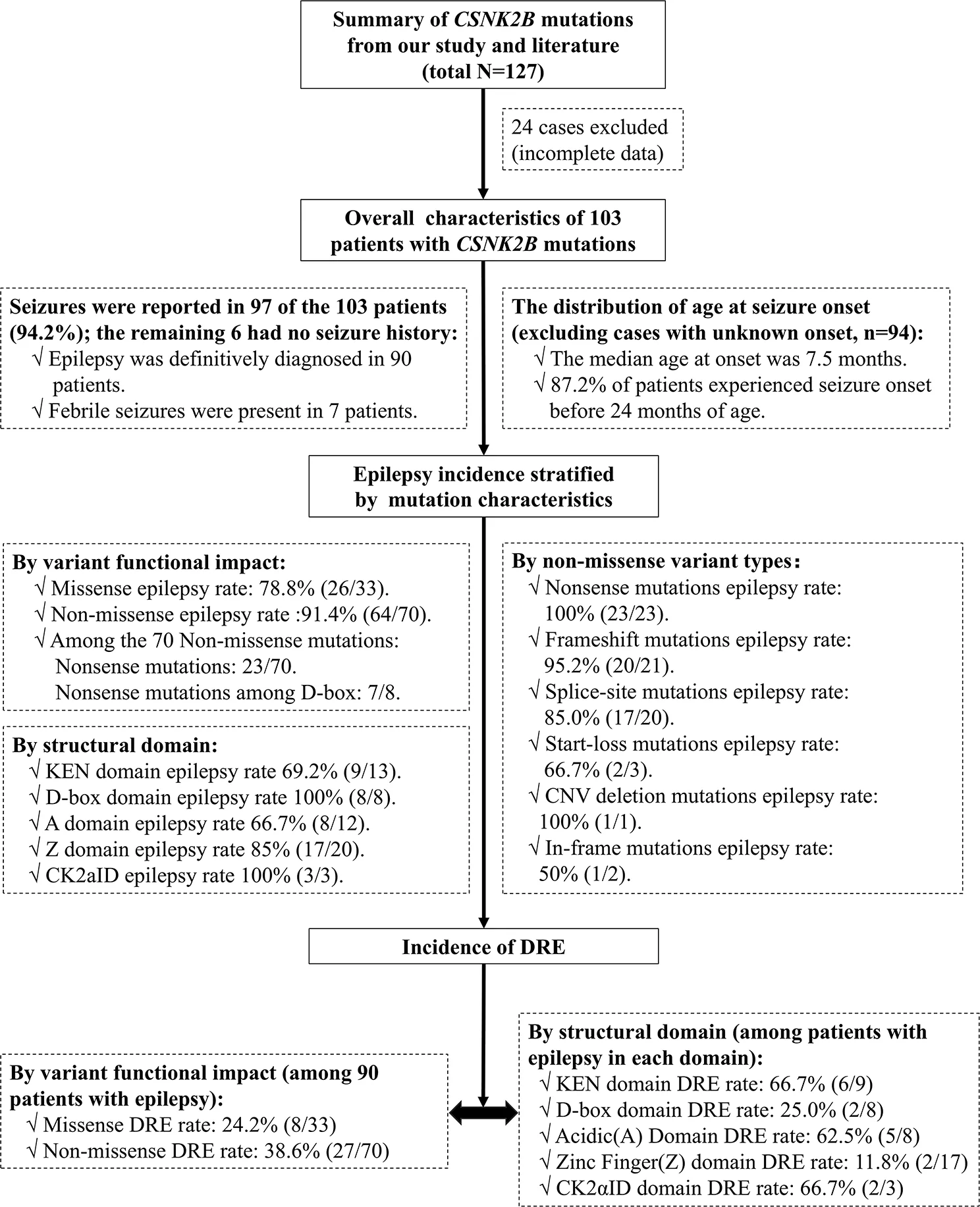
Flowchart of case inclusion for the epilepsy-focused analysis of *CSNK2B* mutations (Three patients were from our center, the remaining cases were curated from previously published reports)

A total of 127 individuals with *CSNK2B* mutations, including the three patients reported in this study, were identified from our family and the published literature. Of these, 103 had sufficiently detailed clinical data for epilepsy-focused analysis, with 93 carrying *de novo* mutations and the remainder having inherited or unknown-origin mutations (Fig. 2 and Supplementary Table 1). In the present family, the origin of the mutations in the father could not be determined because grandparental DNA was unavailable.

Seizures were the most significant clinical feature across the collected cases, with 94.2% of patients (97/103) having a history of seizures. Epilepsy was ultimately diagnosed in 87.4% of patients (90/103), while 6.8% (7/103) had febrile seizures. Seizure type was clearly recorded in 85 cases. Generalized tonic-clonic seizures (GTCS) were the most common (*n* = 49), followed by focal seizures (*n* = 26), myoclonic seizures (*n* = 17), and absence seizures (*n* = 12). A single seizure type was reported in 72.9% of cases (62/85), whereas 27.1% (23/85) had more than one seizure type.

**Fig. 3:**
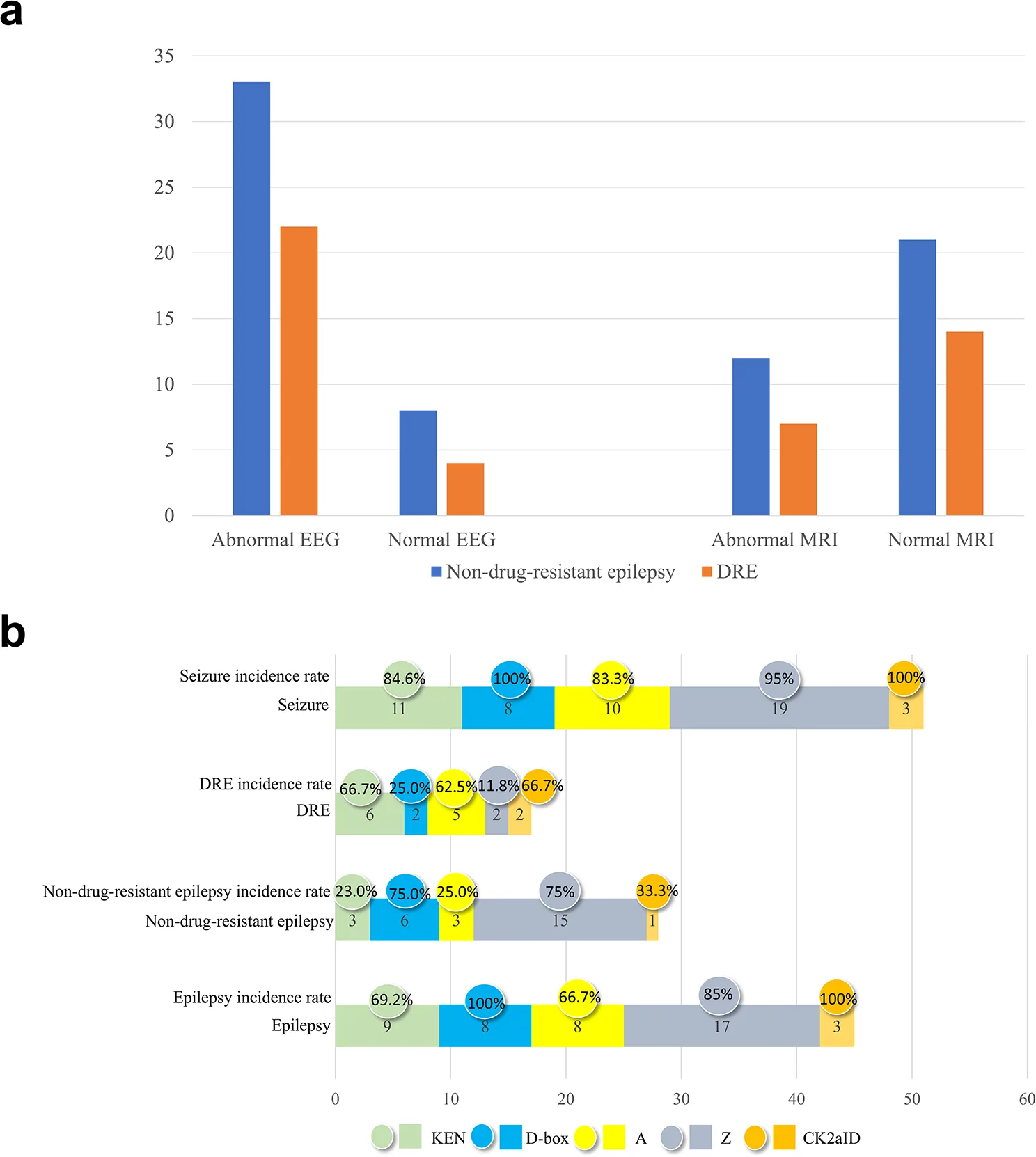
Mutations and epilepsy phenotype data. **a**. Distribution of non-drug‑resistant epilepsy and DRE identified by abnormal/normal EEG and brain MRI findings. **b**. Distribution of seizure, epilepsy, and DRE incidence across different *CSNK2B* structural domains. Squares represent patient counts for epilepsy, non-drug-resistant epilepsy, DRE, seizures; circles indicate the corresponding incidence rates. Each color corresponds to an individual functional domain

The median age at seizure onset was 7.5 months, and most patients (87.2%, 82/94) developed seizures within 24 months of age. For patients with complete EEG and MRI records (Fig. 3a), EEG abnormalities were common (55/67, 82.1%) and typically consisted of generalized or bilateral multifocal spike-and-wave discharges, often with frontal predominance. By contrast, MRI abnormalities were less frequent (19/54, 35.2%), with delayed myelination being the most common finding. Neither EEG nor MRI findings were disease-specific.

Mutation type analysis (Table 2) showed no significant differences between the missense and non-missense groups in seizure rate (87.9% [29/33] vs. 97.1% [68/70], *P* = 0.082), epilepsy rate (78.8% [26/33] vs. 91.4% [64/70], *P* = 0.109), or DRE rate (24.2% [8/33] vs. 38.6% [27/70], *P* = 0.185). The differences in seizure, epilepsy, and DRE rates among nonsense, frameshift, and splice-site mutations were not statistically significant (*P* = 0.313, 0.069, and 0.690, respectively; Fisher-Freeman-Halton exact test, see Table 3). Next, we compared the conservative (*n* = 18) and radical (*n* = 15) missense groups (detailed patient mutations and Grantham score classifications are summarized in Table 4). Epilepsy rates were 72.2% vs. 73.3% and DRE rates 22.2% vs. 26.7%, with no significant differences (*P* = 1.000 and *P* = 0.714, respectively; Fisher-Freeman-Halton exact test).

**Table 2:** Incidence of epilepsy and DRE in missense and non-missense mutation groups

Mutation type	Total	Seizure (%)	Epilepsy (%)	DRE (%)
Missense	33	29 (87.9)	26 (78.8)	8 (24.2)
Non-missense	70	68 (97.1)	64 (91.4)	27 (38.6)
Total	103	97 (94.2)	90 (87.4)	35 (34.0)

* The differences in seizure, epilepsy, and DRE rates between the two groups were not statistically significant (*P* = 0.082, 0.109, and 0.185, respectively; Fisher’s exact test)

**Table 3:** Incidence of seizure, epilepsy, and DRE across non-missense mutation types

Mutation	Total	Seizure (%)	Epilepsy (%)	DRE (%)
Nonsense	23	23 (100.0)	23 (100.0)	7 (30.4)
Frameshift Splice-site Start-loss CNV deletion In-frame	21 20 3 1 2	21 (100.0) 19 (95.0) 2 (66.7) 1 (100.0) 2 (100.0)	20 (95.2) 17 (85.0) 2 (66.7) 1 (100.0) 1 (50.0)	9 (42.9) 8 (40.0) 1 (33.3) 1 (100.0) 1 (50.0)
Total	70	68 (97.1)	64 (91.4)	27 (38.6)

* The differences in seizure, epilepsy, and DRE rates among nonsense, frameshift, and splice-site mutations were not statistically significant (*P* = 0.313, 0.069, and 0.690, respectively; Fisher-Freeman-Halton exact test). Start-loss, CNV deletion, and in-frame mutations were not included in the statistical comparison owing to their very small sample sizes

**Table 4:** Missense mutations and grantham score classifications

Patient ID	Mutation Type	Amino Acid Change	Grantham score	Classification	Epilepsy
9	Missense	p.(Asp32Tyr)	160	Radical	Y**#**
10	Missense	p.(Asp32Asn)	23	Conservative	**/**
11–12	Missense	p.(Asp32Asn)	23	Conservative	Y**#**
13	Missense	p.(Asp32Asn)	23	Conservative	Y
14	Missense	p.(Asp32Asn)	23	Conservative	**/**
15	Missense	p.(Asp32His)	81	Moderately conservative	Y
16	Missense	p.(Phe34Ser)	155	Radical	**/**
17	Missense	p.(Asn35Lys)	94	Moderately conservative	**/**
18–19	Missense	p.(Leu39Arg)	102	Moderately radical	Y**#**
28	Missense	p.(Leu50Pro)	98	Moderately conservative	Y
30	Missense	p.(Glu77Lys)	56	Moderately conservative	**/**
35	Missense	p.(Arg86Cys)	180	Radical	Y
36	Missense	p.(Thr90Pro)	38	Conservative	Y
38	Missense	p.(Met97Ile)	10	Conservative	Y**#**
42	Missense	p.(Phe106Val)	50	Conservative	**/**
43	Missense	p.(Cys109Arg)	180	Radical	Y
44	Missense	p.(Arg111His)	29	Conservative	Y
45–47	Missense	p.(Arg111Pro)	103	Moderately radical	Y
48	Missense	p.(Arg111Pro)	103	Moderately radical	**/**
50	Missense	p.(Cys137Arg)	180	Radical	Y
51	Missense	p.(Cys137Gly)	159	Radical	Y
52–53	Missense	p.(Cys137Phe)	205	Radical	Y
54	Missense	p.(His165Arg)	29	Conservative	Y
55	Missense	p.(His165Arg)	29	Conservative	Y**#**
56	Missense	p.(His165Arg)	29	Conservative	Y
57	Missense	p.(Met166Arg)	91	Moderately conservative	Y
58	Missense	p.(Arg186Lys)	26	Conservative	Y
59	Missense	p.(Leu187Arg)	102	Moderately radical	Y**#**

*Grantham scores were obtained directly from Table 2 of Grantham (1974) [34], which quantifies the physicochemical differences between the original and substituted amino acids in terms of composition, polarity, and molecular volume. Based on the Grantham distance, mutations were classified into four categories according to Pekal and Balatsky (2023) [35]: conservative (0–50), moderately conservative (51–100), moderately radical (101–150), and radical (≥ 151). Higher scores indicate greater physicochemical differences between the amino acid pair and a higher likelihood of deleterious effects on protein function. Y**#**: drug-resistant epilepsy (DRE) or refractory status epilepticus (RSE); Y: presence of epilepsy; “**/”**: absence of epilepsy. Based on Grantham scores, missense mutations were classified as conservative (conservative/moderately conservative) or radical (moderately radical/radical). No significant differences in epilepsy or DRE rates between groups (*P* = 1.000 and *P =* 0.714, Fisher-Freeman-Halton exact test). Given the small sample, these results are descriptive and need confirmation in larger studies

**Fig. 4:**
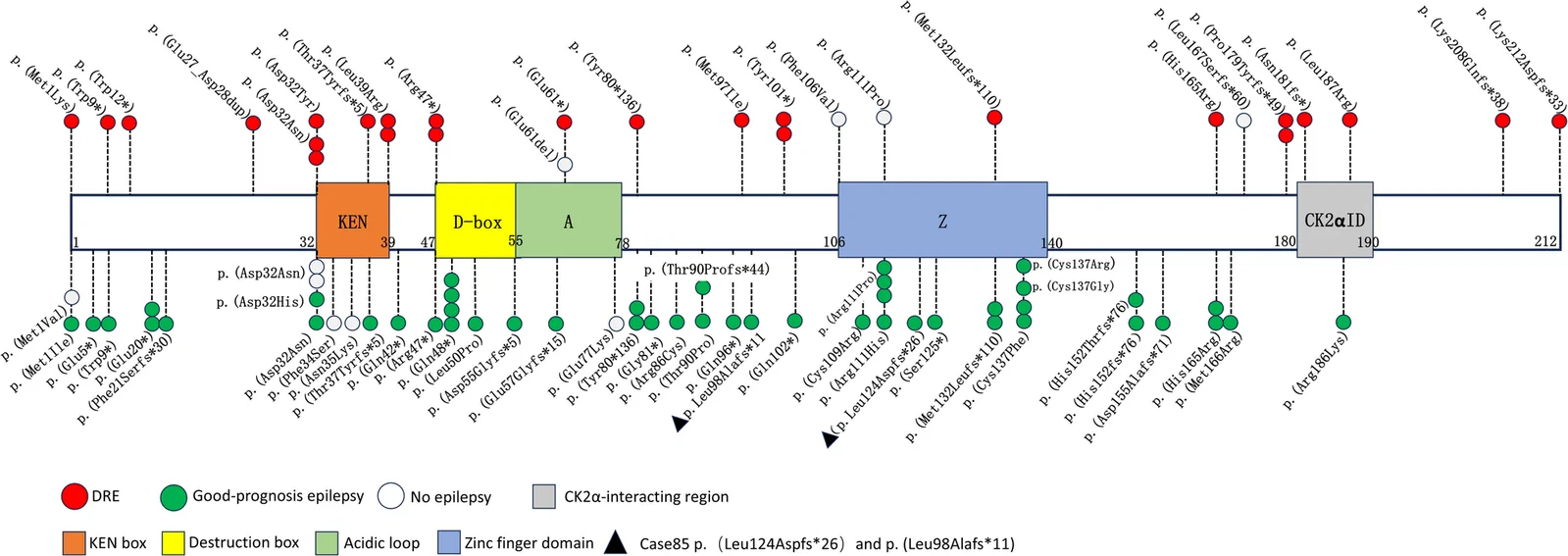
Map of *CSNK2B* mutations and associated epilepsy phenotypes. This map updates previous published *CSNK2B* mutational profiles by incorporating nearly all reported cases and our novel p. Tyr80* mutation case. Meanwhile, it also integrates epilepsy severity (non-epilepsy, non-drug-resistant epilepsy, and DRE) along the protein sequence

When mutations were analyzed according to protein location (Fig. 4; Table 5), the epilepsy rate was significantly higher in patients with mutations located outside annotated canonical domains than in those with mutations within annotated canonical domains (95.7% [44/46] vs. 80.4% [45/56], *P* = 0.034). Outside annotated canonical domains, mutations were mainly distributed in the N-terminal region (14 cases) and linker region (25 cases), with fewer mutations in the C-terminal region (7 cases). Within annotated canonical domains, epilepsy penetrance was 100% in the D-box and CK2α-interaction domains, 85.0% in the Z-domains, 69.2% in the KEN-box, and 66.7% in the acidic region. DRE frequencies were highest in the KEN-box and CK2α-interaction domains (both 66.7%), followed by the acidic region (62.5%), and were lower in the D-box (25.0%) and Z-domains (11.8%) (Fig. 3b). To provide a phenotype-oriented overview of mutation distribution, we mapped previously reported and newly identified *CSNK2B* mutations onto the protein domain schematic, grouped them by epilepsy phenotype (non-epilepsy, non-drug‑resistant epilepsy, and DRE), and summarized the results in Fig. 4. In addition, we observed that most nonsense mutations were located outside the five annotated functional domains (14/23), while seven of the eight nonsense mutations within annotated domains were clustered in the D-box.

**Table 5:** Incidence of seizure and epilepsy between domain and non-domain mutations

Mutation site	Total	Seizures (%)	Epilepsy (%)
Domain	56	51 (91.1)	45 (80.4)
Non-domain	46	45 (97.8)	44 (95.7)
Total	102	96 (94.1)	89 (87.3)

*One case with a CNV deletion mutation was excluded from the analysis. The difference in epilepsy rates between the two groups was statistically significant (*P* = 0.034, Fisher’s exact test)

## Discussion

Here, we reported a novel pathogenic nonsense mutation, c.240T > G, p. Tyr80*, in three affected individuals from a family with early-onset seizures. The mutation is absent from population databases, shows perfect familial co‑segregation, and is predicted to be highly deleterious. Intriguingly, despite modest genomic conservation scores (GERP + + RS = 2.19; phyloP = 0.448), the p. Tyr80* residue is highly conserved at the protein level across multiple species, highlighting its functional importance. Taken together with the known role of CK2β in neurodevelopment and neuronal excitability [22], these data supported the interpretation that this mutation was disease-causing in this family.

Our epilepsy-focused synthesis further confirms that epilepsy remains the most prominent phenotype, with seizures typically beginning within the first 2 years of life, and exhibiting substantial heterogeneity in seizure type, treatment response, and developmental outcome. These findings are consistent with previous studies suggesting that broad mutation grouping alone has limited value in predicting epilepsy severity in *CSNK2B*-related disorders [5, 23, 24].

Nonsense mutations are associated with relatively high rates of seizures and epilepsy. Previous research has suggested that haploinsufficiency may contribute to the pathogenesis of some *CSNK2B*-related disorders, but it should be noted that different mutation types have direct effects on protein products and function [25]. In *CSNK2B*, some frameshift mutations might have abolished the canonical stop codon and created a new downstream termination site, making them less likely to be recognized by canonical nonsense-mediated mRNA decay (NMD) and thereby allowing production of aberrant protein products [7, 26, 27]. It is well established that premature termination codons located within the last exon or within 55 bp of the last exon-exon junction can escape degradation by canonical NMD [28]. Notably, the patient carrying p. Leu167Serfs*60 had febrile seizures but no epilepsy, and in vitro studies showed reduced abundance of the mutant protein [29]. This mutation is located in exon 5 of *CSNK2B* and is predicted to trigger NMD. Among our summarized cases, only one mutation, p. Lys208Glnfs*38, was located within the NMD-escape region and presented with DRE. Since this mutation resides in a more downstream exon, it might theoretically escape NMD and produce a truncated aberrant protein, which could contribute to a more severe phenotype. Future validation using patient samples is required to verify the NMD-escape effect of the mutant transcript and clarify the functional impact of the resulting truncated protein.

In missense mutations, conservative and radical groups did not differ in epilepsy or DRE rates (*P* = 1.000 and *P* = 0.714, Fisher-Freeman-Halton exact test), indicating no clear association with clinical phenotypes. Grantham scores just reflect static physicochemical differences and are unlikely to fully predict complex in vivo outcomes. Our analysis is only predictive and exploratory, lacking experimental support. However, this result did not fully align with functional data from specific mutations: the CK2 holoenzyme carrying p. Asp32His (score 81, moderately conservative) showed markedly reduced activity, whereas p. Asp32Asn (score 23, conservative) did not, suggesting that the effect on phosphorylation generally paralleled the Grantham scores [10]. In our cases, the single patient with p. Asp32His had epilepsy but not DRE. Among the five patients with p. Asp32Asn, three had epilepsy and two were DRE. This contrast might suggest that other factors also contribute. Interestingly, even patients sharing the same p. Asp32Asn mutation showed different phenotypes. This points to genetic background or modifier genes as possible modulators of penetrance. It has been proposed that some missense mutations might have retained residual function, whereas mutations affecting critical residues might have impaired CK2 holoenzyme activity through dominant-negative effects [14, 30]. Whether dominant‑negative effects are involved and how much residual function missense mutations retain await experimental verification.

Current evidence suggests that the CK2α-interaction domain directly mediated CK2β-CK2α binding, whereas residues at positions 55, 57, and 59–64 within the acidic region were critical for regulating CK2 holoenzyme activity, and alanine substitutions at these sites caused abnormal enzyme activation [31, 32]. In line with these previous observations, mutations in these regions might be associated with more severe phenotypes, whereas zinc finger domain mutations were more often linked to relatively mild and more controllable epilepsy [7]. However, the number of reported cases involving the CK2α-interaction domain remained limited, and conclusions regarding this region require further validation in larger cohorts. Further analysis showed that mutations outside known functional domains had a higher epilepsy penetrance than those within domains (95.7% vs. 80.4%, *P* = 0.034), and these mutations were mainly clustered in the N‑terminal region and linker region. These regions may contain a conserved helical groove that may contribute to protein–protein interactions within the CK2β dimer [33]. In addition, although the KEN box and D-box functioned as APC/C-recognized degradation motifs [26], they showed different clinical associations in our study. All D-box mutations were accompanied by epilepsy, but the DRE rate was relatively low, whereas KEN box mutations were more often associated with DRE. At the same time, we observed that D-box mutations were predominantly nonsense mutations, whereas KEN box mutations were more often missense mutations or other alterations that could partially preserve protein structure. We speculated that this clinical difference might have reflected regional differences in mutation-type distribution. D-box mutations were more likely to act through protein truncation and haploinsufficiency, whereas KEN box mutations might have produced aberrant proteins capable of participating in complex assembly and exacerbating holoenzyme dysfunction through dominant-negative effects, thereby increasing DRE risk. These findings suggested that, beyond mutation location, the distribution of mutations across different domains varies, and this variation may affect epilepsy severity.

In summary, we need to consider that truncated proteins trigger NMD to worsen haploinsufficiency or escaping NMD to produce a dominant‑negative effect, as well as the conservation of missense mutations, all require experimental validation.

This study had several limitations. First, it combined one newly reported family with a retrospective dataset from the literature, making some degree of error in data collection and extraction unavoidable. Second, repeated case reports and incomplete phenotyping in the rare-disease literature may have affected the accuracy of data integration. Third, no functional studies were performed for c.240T > G, p. (Tyr80*), so mechanistic interpretations should be made cautiously. Fourth, the hypotheses proposed in this study lack experimental verification. The main limitations are the small sizes of several subgroups (e.g., start-loss, CNV deletion, in‑frame) and the literature‑based nature of the dataset, so the results should be considered exploratory and require confirmation in larger cohorts with functional assays.

## Conclusions

Our findings identified a novel pathogenic nonsense mutation in *CSNK2B*, and expanded the phenotypic spectrum of epileptic heterogeneity in *CSNK2B*-related disorders. Consistent with previous studies, grouping by mutation type had limited value in predicting epilepsy severity, whereas exploratory analyses suggested that protein subregional location appeared to be more informative. Furthermore, the distribution of mutation types across different domains showed some variability. These findings require validation in larger cohorts and functional studies.

## Supplementary Information

Below is the link to the electronic supplementary material.

Supplementary Material 1

## Data Availability

All data supporting the findings of this study are available within the paper and its Supplementary Information.

## Abbreviations

ACMG: American College of Medical Genetics and Genomics
ASMs: Antiseizure medications
CADD: Combined Annotation Dependent Depletion
CNV: Copy number variation
DEE: Developmental and epileptic encephalopathy
DRE: Drug-resistant epilepsy
EEG: Electroencephalography
GERP++: Genomic Evolutionary Rate Profiling
GTCS: Generalized tonic-clonic seizures
HGMD: Human Gene Mutation Database
ILAE: International League Against Epilepsy
LEV: Levetiracetam
LOF: Loss-of-function
MRI: Magnetic resonance imaging
NMD: Nonsense-mediated mRNA decay
OXC: Oxcarbazepine
phyloP: Phylogenetic *P*-value
VPA: Valproic acid
